# Patient characteristics and interventions at a trauma stabilization point in Gaza– a retrospective analysis

**DOI:** 10.1186/s13031-026-00795-0

**Published:** 2026-05-05

**Authors:** Wessam El Ghoul, Hareen De Silva, Samar Al-Hajj, Jonas Zimmerman, Marta Caviglia, Johan von Schreeb, Luca Pigozzi, Flavio Salio, Luca Ragazzoni, Martin Gerdin Wärnberg

**Affiliations:** 1 Department of Global Public Health, Karolinska Institutet, Stockholm, Sweden; 2 Department of Emergency Medicine, Karolinska University Hospital, Stockholm, Sweden; 3 Cadus e.V, Berlin, Germany; 4 Department of Epidemiology and Biostatistics, Faculty of Health Sciences, American University of Beirut, Beirut, Lebanon; 5 Centre for Disaster Medicine, University of Gothenburg, Gothenburg, Sweden; 6 CRIMEDIM - Center for Research and Training in Disaster Medicine, Humanitarian Aid and Global Health, Università del Piemonte Orientale, Novara, Italy; 7 Department of Translational Medicine, Università del Piemonte Orientale, Novara, Italy; 8 World Health Organization, OPT (Occupied Palestinian Territory), Gaza, Palestine; 9 World Health Organization, Geneva, Switzerland; 10 Department for Sustainable Development and Ecological Transition, Università del Piemonte Orientale, Vercelli, Italy; 11 Department of Perioperative Medicine and Intensive Care, Karolinska University Hospital, Stockholm, Sweden; 12 K9 Global folkhälsa, K9 GPH von Schreeb, Karolinska Institutet, Stockholm, 171 77 Sweden

**Keywords:** Trauma stabilization point, Prehospital care, Conflict settings, Gaza war, Injury patterns, Triage, Emergency medical response.

## Abstract

**Background:**

Timely prehospital care is essential in preventing trauma‑related deaths in conflict settings, yet civilian systems often lack the capacity for rapid stabilization and evacuation. Trauma Stabilization Points (TSPs), adapted from military models, have been introduced in Gaza to provide forward‑deployed triage and stabilization. This study describes patient characteristics, clinical presentations, interventions, and outcomes at a TSP operating in Khan Younis, Gaza, in 2024 during the ongoing war.

**Methods:**

We conducted a retrospective cross‑sectional analysis of routinely collected patient‑level data from a TSP operated by the Palestinian Red Crescent Society with support from the WHO and an international emergency medical team. All patients presenting between 12 February and 28 April 2024 were included. Descriptive statistics and logistic regression assessed associations between patient characteristics, injury severity, interventions, and referral outcomes.

**Results:**

1,928 patients were entered into the dataset. Non‑traumatic conditions accounted for 53% of attendances, while 47% were trauma‑related. Most patients (94.8%) were stable, as defined by the Interagency Integrated Triage Tool (IITT) on arrival. A total of 3482 interventions were recorded, dominated by basic care (86%). Overall, 80.6% of patients were discharged, 19.3% referred to still-functioning hospitals, and 0.1% died at the TSP. Clinical instability, fractures, and penetrating injuries were significantly associated with referral, while basic interventions (e.g., wound suturing, antipyretics) predicted discharge.

**Conclusions:**

Despite operating in a high‑intensity conflict, the TSP primarily functioned as a triage and primary‑care access point rather than a trauma‑focused facility. The dominance of on-site treatment and same-day discharge suggest the TSP offloaded some non-urgent patients from overwhelmed hospitals and likely contributed to reducing consumption of limited ambulance resources.

## Background

Studies suggest that up to 60% of traumatic deaths in conflict settings may be preventable through timely interventions addressing external hemorrhage, airway obstruction, and other treatable causes [1, 2]. The prehospital stage, which includes all care provided until arrival at a hospital, is critical for minimizing delays in administering these interventions [3, 4]. Military trauma systems reduce mortality through rapid evacuation and the deployment of forward surgical teams; however, these systems are resource-intensive, limiting their transferability to civilian trauma care in armed conflicts [5, 6]. Given the growing number of civilians affected by conflict, there is a need to develop context-adapted prehospital care that is cost-effective and feasible [7–9]. 

To address this need, governmental health authorities and international humanitarian actors, under WHO guidance, have introduced the concept of Trauma Stabilization Points (TSPs), also referred to as “Medical Access Points” or “Triage Points” [10]. Adapted from military systems, they are temporary, forward-deployed medical sites designed to deliver triage and stabilization [11–13]. Previous deployments of TSPs in Iraq (2016–2017) and Gaza (2018–2019) demonstrated operational feasibility and potential clinical benefits, though impact on mortality reduction remains uncertain [13–16]. In Mosul, TSPs operated within a broader emergency medical system, with accessible hospitals and some degree of operational safety, including partial embedding with military units [13, 15]. 

Several TSPs have been operational in Gaza since the start of the war in October 2023 [17]. This study analyses demographics, injury mechanisms, severity, interventions, and outcomes using data compiled from one TSP operating during the war in Gaza in 2024.

## Methods

### Study design

This is a retrospective cross-sectional descriptive analysis of routinely collected patient-level data from a TSP in Khan Younis, Gaza, in 2024 during the ongoing war.

### Setting

The first TSP of the conflict was established in February 2024 by the Palestinian Red Crescent Society (PRCS), with support from WHO and an international Emergency Medical Team (EMT) type 1, deployed by Cadus e.V., a humanitarian organization specializing in providing prehospital care in conflict settings. By that time, Israeli attacks had reportedly resulted in at least 30,000 deaths in Gaza, causing widespread destruction of the pre-existing healthcare sector [18–20]. 

CADUS partly staffed the TSP with one mobile team consisting of 2–6 doctors with nurses and paramedics, classified according to WHO EMT minimum standard [21]. The medical team consisted of residents and attendings/specialists in emergency medicine and internal medicine. The team operated daily in collaboration with PRCS personnel, who also provided ambulances. For safety reasons, the facility operated during daylight hours, from 08:00 to 16:30. The EMT could perform triage and all immediate lifesaving interventions, including intubation, chest drain insertion, and intravenous fluid resuscitation.

All cases were triaged on arrival using the WHO IITT and classified stable (those triaged yellow or green) or as unstable/deteriorating (those triaged red) [22]. The operational protocol aimed for stabilization of the critically injured within 15 min, followed by immediate evacuation to a hospital.

**Fig. 1:**
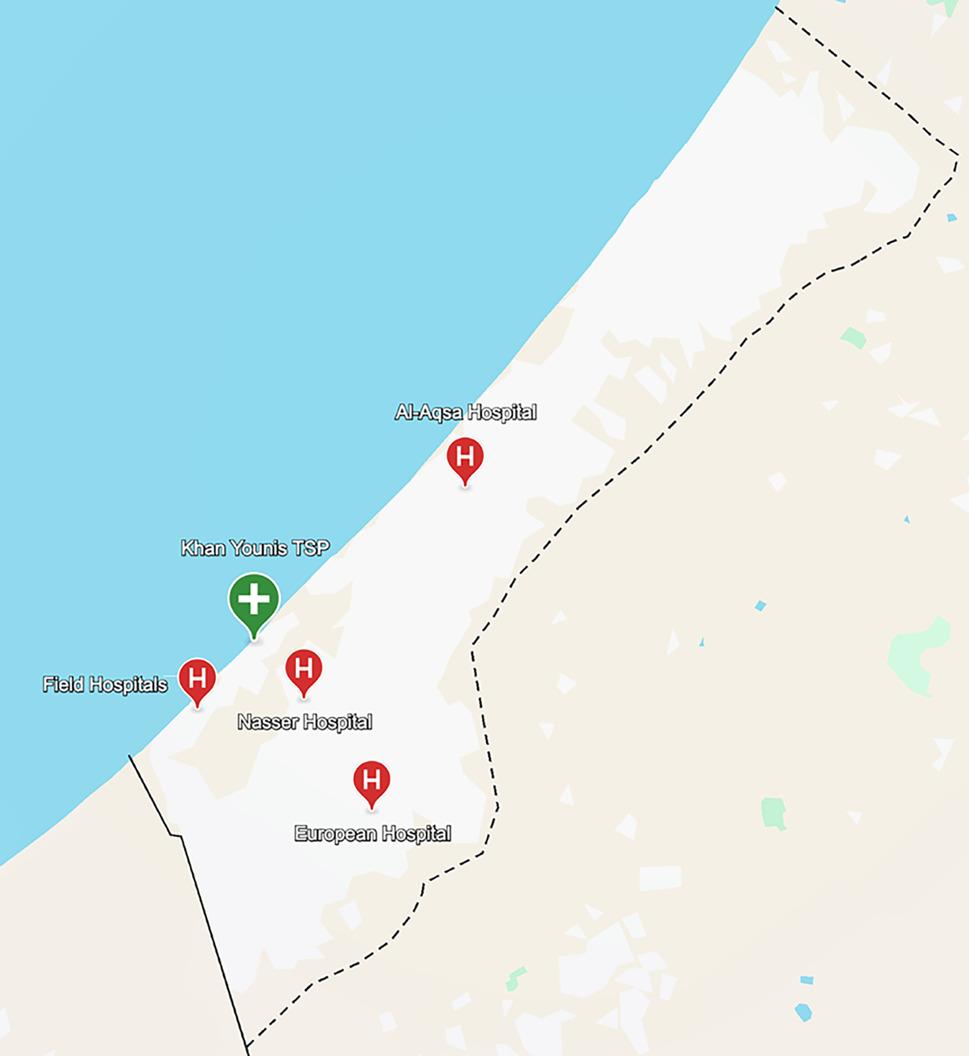
Map of Gaza with location of Khan Younis TSP and main receiving hospitals

The location of the TSP was strategically selected by the WHO, PRCS, and the Palestinian Ministry of Health to serve as an immediate point of care for casualties during the Israeli military offensive in Khan Younis, which rendered the local major tertiary facility, Nasser Medical Complex, non-operational [23]. The TSP was set up in tents at a football field and located along an evacuation route from major population centers (see Fig. 1), in proximity to the main north–south movement axis. This offered two evacuation options for the rapid transport of casualties: northwards to Al-Aqsa Hospital in the Middle Area, or southwards to field hospitals. The Khan Younis TSP closed in April 2024 and relocated to Rafah to manage casualties from another military offensive. In April 2024, Nasser Medical Complex resumed its activities, further limiting the need for a TSP in that location.

### Participants

All patients recorded at the TSP between 12 February and 28 April 2024 were included, except for those dead on arrival.

### Variables

Routine patient data variables collected included age group, sex, injury types (penetrating, blunt, burns, amputations, fractures, and lacerations), non-traumatic diagnoses, interventions administered, and outcomes (discharged, referred, deceased). Clinical status was recorded according to the IITT, with those triaged green or yellow defined as stable and those triaged red defined as unstable.

The dataset lacked a variable for injury severity or proxy variables for severe injuries such as hypotension, tachycardia or a low Glasgow Coma Scale. We therefore operationalized severe injury as any case with penetrating or blunt trauma, burns, amputations, or fractures to the head, neck, face, thorax, abdomen, or pelvis. This aligns with the Abbreviated Injury Scale as indicating a severe injury [24]. 

No data were available on pre-arrival interventions or post-referral outcomes.

### Data sources

Data were collected by Cadus e.V. clinicians trained on using the WHO Minimum Data Set via the KoboToolbox software package [25]. Initial documentation was done on paper and later digitized. Once internet connectivity was available, the data were automatically uploaded to the data server. All uploaded data were anonymized to ensure patient confidentiality.

### Quantitative variables

Age was categorized into predefined groups (< 5, 5–17, 18–49, 50–59, > 60) as per Cadus predetermined reporting standard. Injury severity and number of interventions were treated as continuous variables. Binary indicators were used for injury types and clinical status.

### Statistical methods

Analyses were conducted using R version 4.5. via RStudio version 2025.05.0 + 496 running on macOS. Descriptive statistics summarized sample characteristics. Binary logistic regression assessed associations between patient characteristics and outcomes. Separate logistic regression models were used to evaluate the relationship between injury severity and individual interventions, and associations between outcome and key variables. Odds ratio, 95% CIs, and p-values were calculated. A *p* < 0.05 indicated statistical significance.

## Results

A total of 2001 patient visits were recorded at the TSP between February 12th and April 28th, 2024. Of these, 73 patients were classified as dead on arrival and therefore not included in further analysis. The remaining 1928 patients had a documented outcome.

### Participant characteristics

**Fig. 2:**
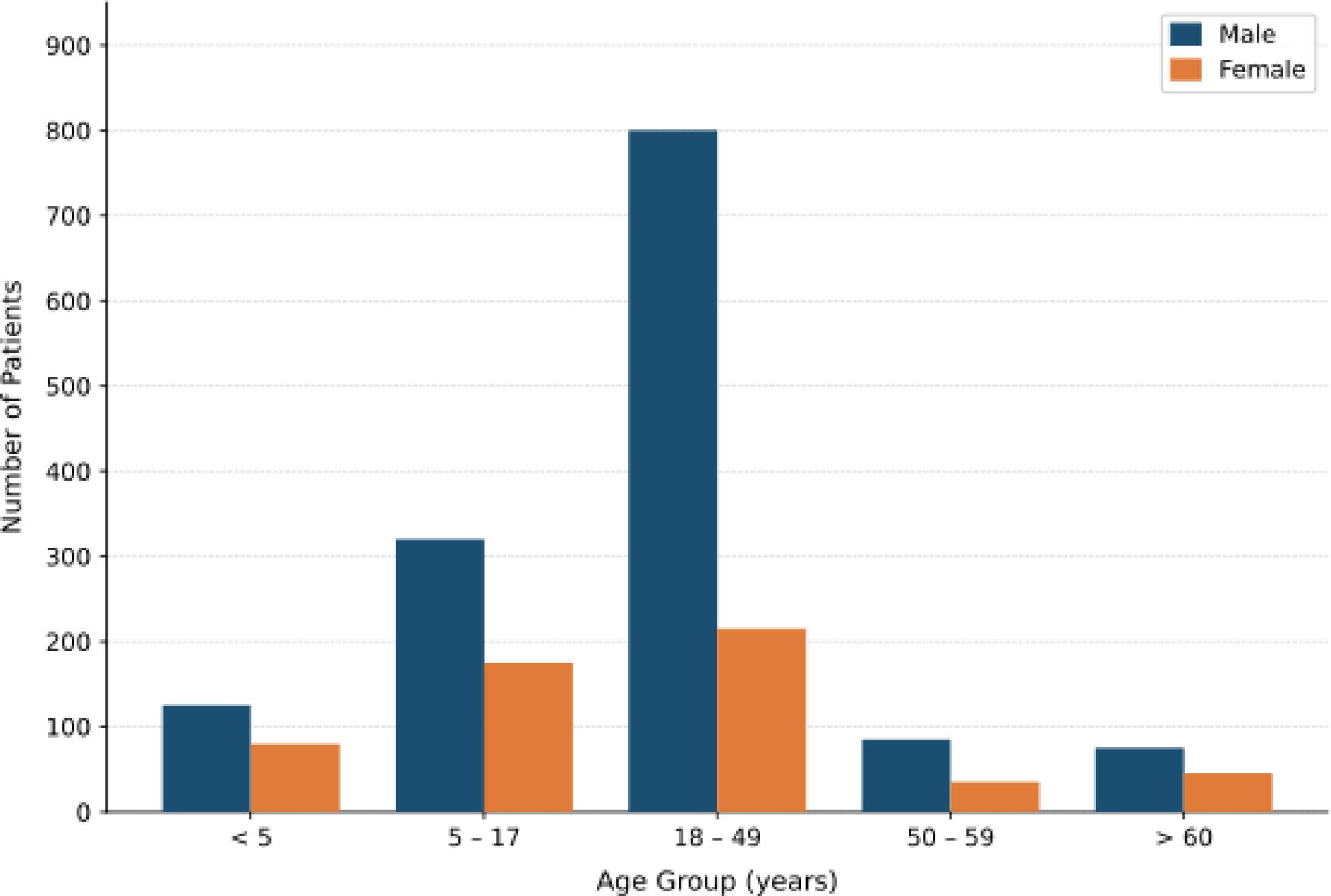
Age groups and gender composition

Most patients were in the 18–49 age group (52.6%), followed by children aged 5–17 years (26.0%) (Table 1). Children under five accounted for 10.5% of the sample, while adults aged 50–59 and those ≥ 60 years represented 5.5% and 5.4%, respectively. Males were overrepresented across all age groups, particularly in the 18–49 age group, where they constituted 78.4% of patients (Fig. 2).

Clinical status on arrival was recorded for all patients (Table 1). Most were stable (94.8%), while 5.2% were classified as unstable or deteriorating.

Non-traumatic medical conditions accounted for 53% of all cases seen at the TSP (Table 2). These included gastrointestinal complaints (13.7%), infectious diseases (6.1%), musculoskeletal issues (5.4%), and respiratory conditions (2.7%). Trauma comprised the remaining 47%, with lacerations (16%), penetrating injuries (12%), and blunt trauma (11%) being the most common. Burns, amputations, and fractures were less frequent. 123 cases (6.4%) of head trauma were recorded, 21 of which were penetrating.

**Fig. 3:**
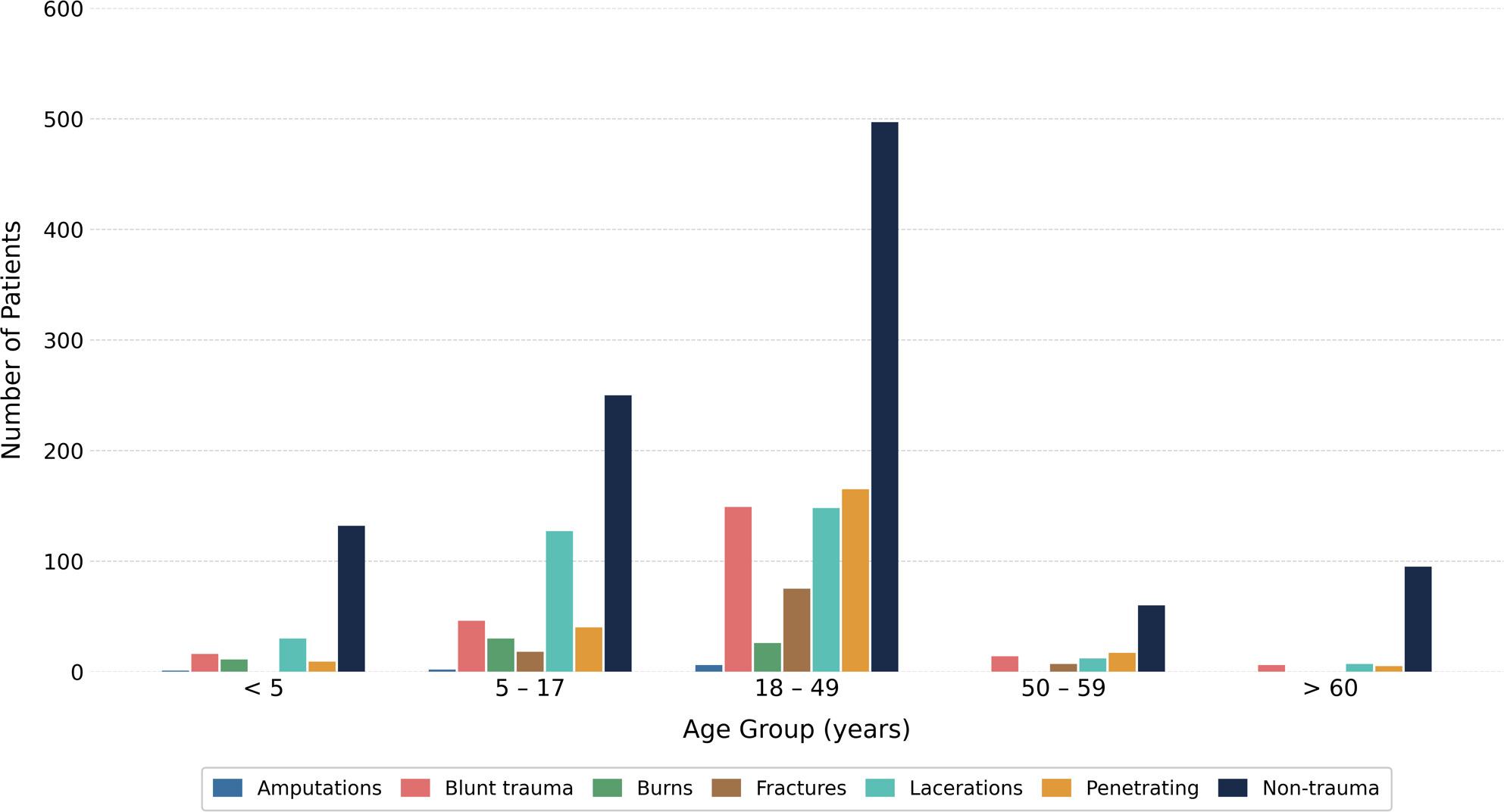
Number of injuries by injury types and age group

Blunt trauma was most frequently seen in adults. Younger age groups had proportionally more penetrating injuries compared to older groups, while elderly patients (> 60 years) exhibited a higher share of non-traumatic causes for attendance (Fig. 3 ).

**Table 1:** Summary of key observations

Variable	Category	Count (%)
**Age groups**	Under 5 years	202 (10·5)
	5–17 years	502 (26·0)
	18–49 years	1012 (52·6)
	50–59 years	107 (5·5)
	60 + years	105 (5·4)
**Clinical status**	Stable	1827 (94·8)
	Deteriorating	99 (5·2)
	Severe injury	181 (9·4)
**Reason for attendance**	Medical	1023 (53)
	Trauma	933 (47)
**Interventions**	Basic care	2980 (86)
	Monitoring	93 (3)
	Advanced care	146 (11·6)
	Unspecified	263 (13)
**Outcome**	Discharged	1554 (80·6)
	Referred	372 (19·3)
	Died at TSP	2 (0·1)

**Table 2:** Breakdown of injury types and non-traumatic causes

Cause	Type	Count (%)
**Trauma**	All trauma	933* (47)
	Lacerations	307 (16)
	Penetrating Injuries	230 (12)
	Blunt Injuries	219 (11)
	Fractures	104 (5)
	Burns	65 (3)
	Amputations	8 (< 1)
**Non-trauma**	All non-trauma	1023 (53)
	Gastrointestinal	264 (13·7)
	Unspecified non-trauma	183 (9·5)
	Infectious diseases	117 (6·1)
	Musculoskeletal	104 (5·4)
	Respiratory	51 (2·7)
	ENT/ophthalmological	32 (1·7)
	Renal and urogenital	31 (1·6)
	Neurological	28 (1·5)
	Endocrine/metabolic	27 (1·4)
	Dermatological	23 (1·2)
	Cardiovascular	19 (1)
	Mental health	13 (0·7)
	Gynaecological	13 (0·7)
	Cancer	7
	Thrombo-embolic	7
	Haematological	3

*28 traumatic cases had more than one injury recorded

### Outcomes

Of the 1,928 patients included in the analysis, 1,554 (80.6%) were discharged to their place of residence, 372 (19.3%) were referred to a higher-level medical facility, and 2 patients (0.1%) died at the TSP (Table 1). This pattern was observed across all age groups (Table 3).

Majority of stable cases were discharged (82%) while 45% of deteriorating patients were referred.

**Table 3:** Number of referrals and discharges according to age group

Age Group	Discharged (%)	Referred (%)
< 5	159 (78·7)	41 (20·3)
5–17	415 (82·7)	85 (16·9)
18–49	806 (79·5)	208 (20·5)
50–59	86 (80·4)	20 (18·7)
≥ 60	88 (83·8)	18 (17·1)

### Interventions

A total of 3,482 clinical interventions were recorded (Table 4). Basic care interventions dominated, accounting for 86% of all procedures. These included wound care (44.1%), medical advice (21.9%), analgesia (20.2%), and IV access (13.6%). Advanced interventions such as airway management, mechanical ventilation and chest drain insertions were rare (11.6%), in line with the observed low numbers of cases classified as unstable. Monitoring interventions (vital signs and cardiac monitoring) comprised 3%, while 13.5% of interventions were categorized as other. The majority in this category were unspecified or included rare interventions such as assistance with childbirth.

**Table 4:** Number and percentages of all interventions

Category	Intervention	Count (%)
**Advanced**	Sedatives	64 (3·3)
	Synchronised cardioversion	32 (1·7)
	Airway management	14 (0·7)
	Inotropes/vasopressors	6 (0·3)
	Suction	6 (0·3)
	Drain insertion	5 (0·3)
	Endotracheal intubation	4 (0·2)
	Drain management	2 (0·1)
	Mechanical ventilation	2 (0·1)
	Tourniquet application	2 (0·1)
	Cardiopulmonary resuscitation	1 (0·1)
**Basic Care**	Wound care	852 (44·1)
	Medical advice	423 (21·9)
	Analgesia	389 (20·2)
	IV access	263 (13·6)
	Fluid therapy	215 (11·2)
	Antibiotics	212 (11)
	Wound suture	203 (10·5)
	Antipyretic Therapy	144 (7·5)
	Splinting	71 (3·7)
	Warming/cooling	49 (2·5)
	Tranexamic acid	38 (2)
	Suture removal	25 (1·3)
	Oxygen	24 (1·2)
	Opioids	22 (1·1)
	Chest seal application	17 (0·9)
	Nebulisation	16 (0·8)
	Wound packing	13 (0·7)
**Monitoring**	Vitals (HR, BP, SpO2, RR)	57 (3)
	Cardiac monitoring	23 (1·2)
	Sonography	12 (0·6)
**Other**	Unspecified	261 (13·5)
	Psychological First Aid	10 (0·5)
	Personal hygiene	3 (0·2)
	Assistance with childbirth	2 (0·1)

### Severe injuries and referrals

**Fig. 4:**
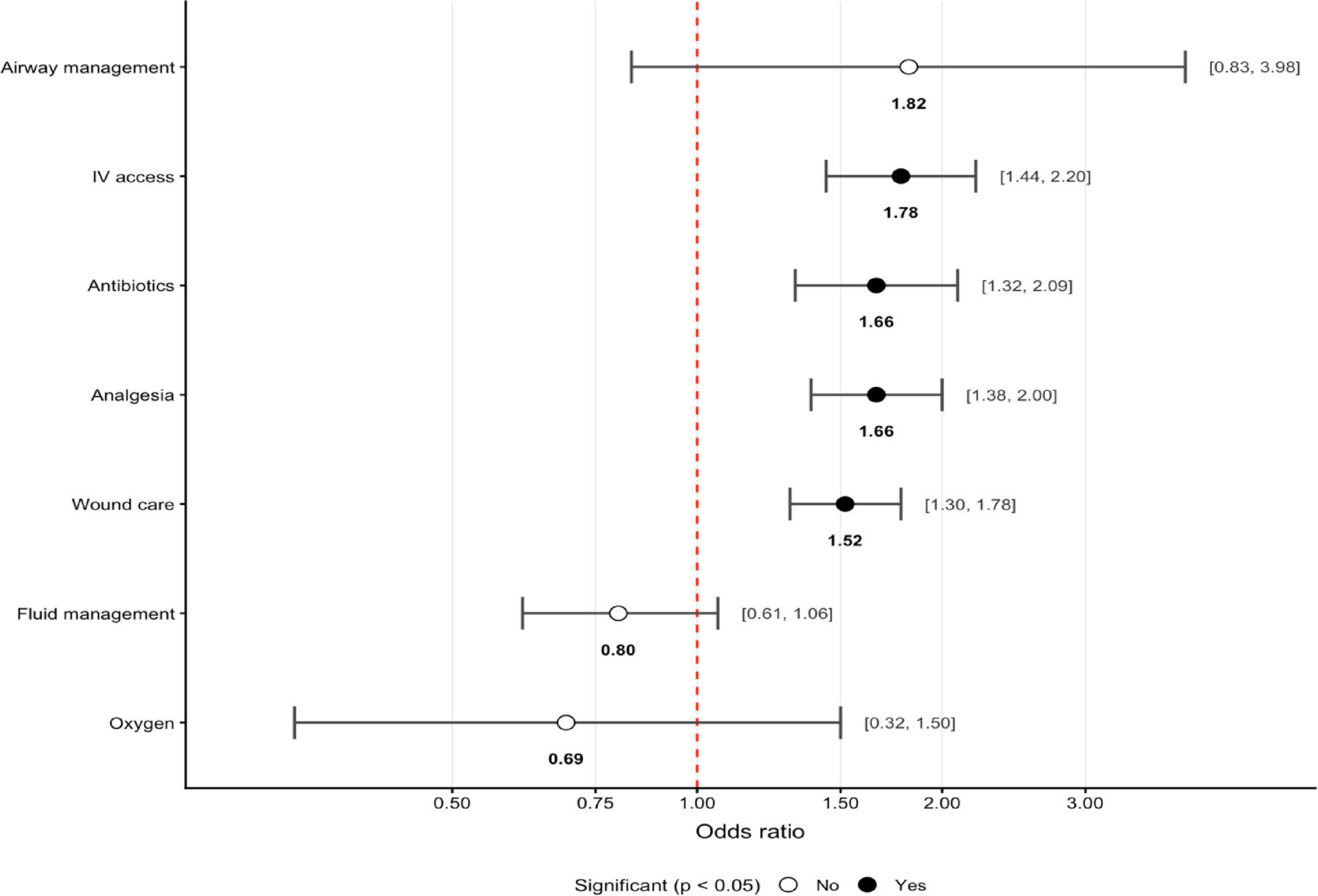
Odds Ratio with 95% CI of Interventions Associated with Severe Injury

Logistic regression identified IV access (OR 1.78; 95% CI 1.44–2.20), antibiotic administration (OR 1.66; 95% CI 1.32–2.09), wound care (OR 1.52; 95% CI 1.30–1.78), and analgesia (OR 1.66; 95% CI 1.38–2.00; all *p* < 0.01) as the interventions most strongly associated with severe injuries (Fig. 4).

**Fig. 5:**
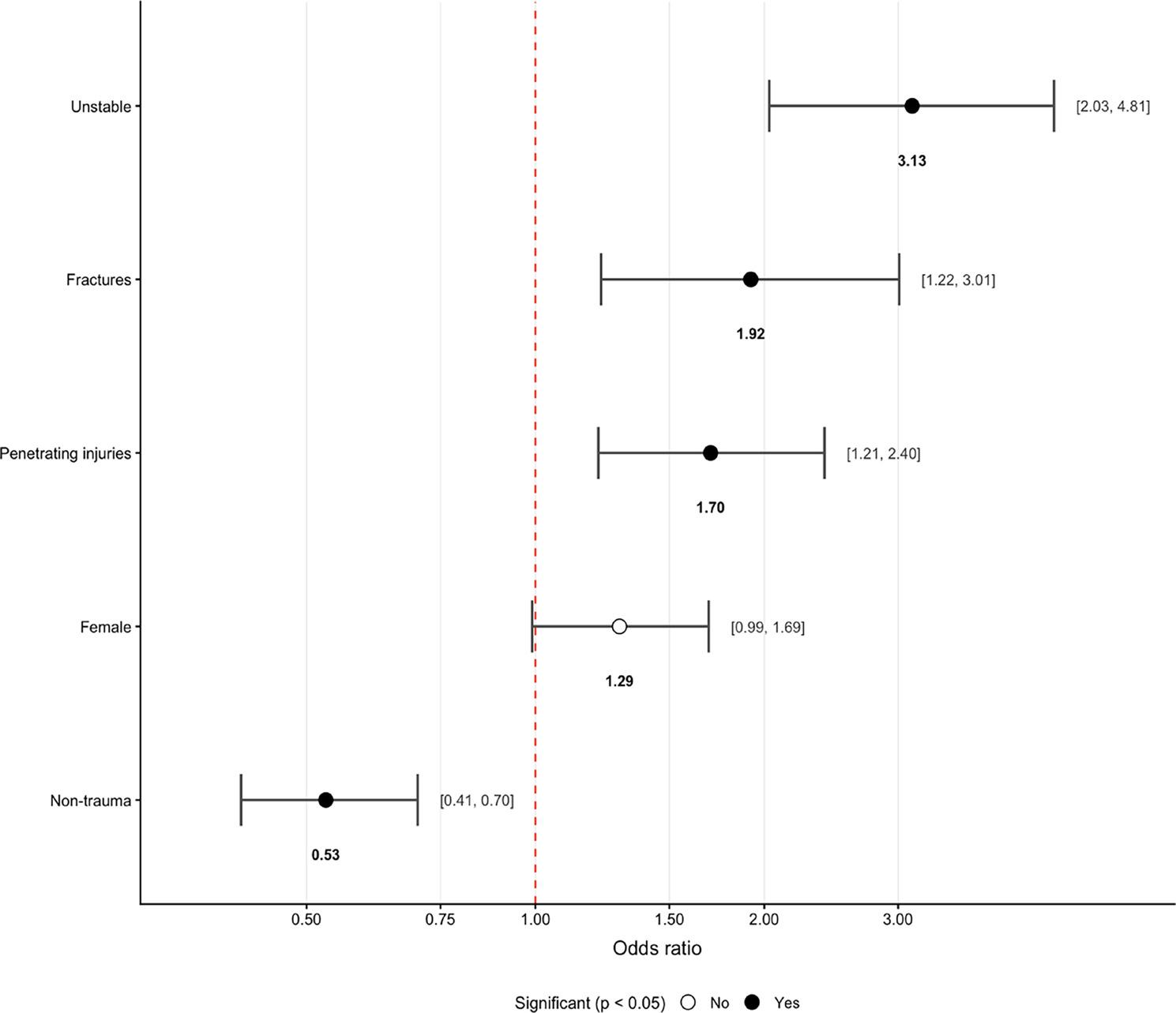
Odds Ratio with 95% CI of Patient Characteristics Associated with Referral

Clinical instability was the strongest predictor of referral to a higher level of care (OR 3.13; 95% CI 2.03–4.81; *p* < 0.001) (Fig. 5), followed by fractures (OR 1.92; 95% CI 1.22–3.01; *p* = 0.005) and penetrating injuries (OR 1.70; 95% CI 1.21–2.40; *p* = 0.002). Non-traumatic medical conditions were associated with a reduced likelihood of referral (OR 0.53; 95% CI 0.41–0.70; *p* < 0.001), while burns, blunt trauma, amputations, and lacerations showed no significant associations.

**Fig. 6:**
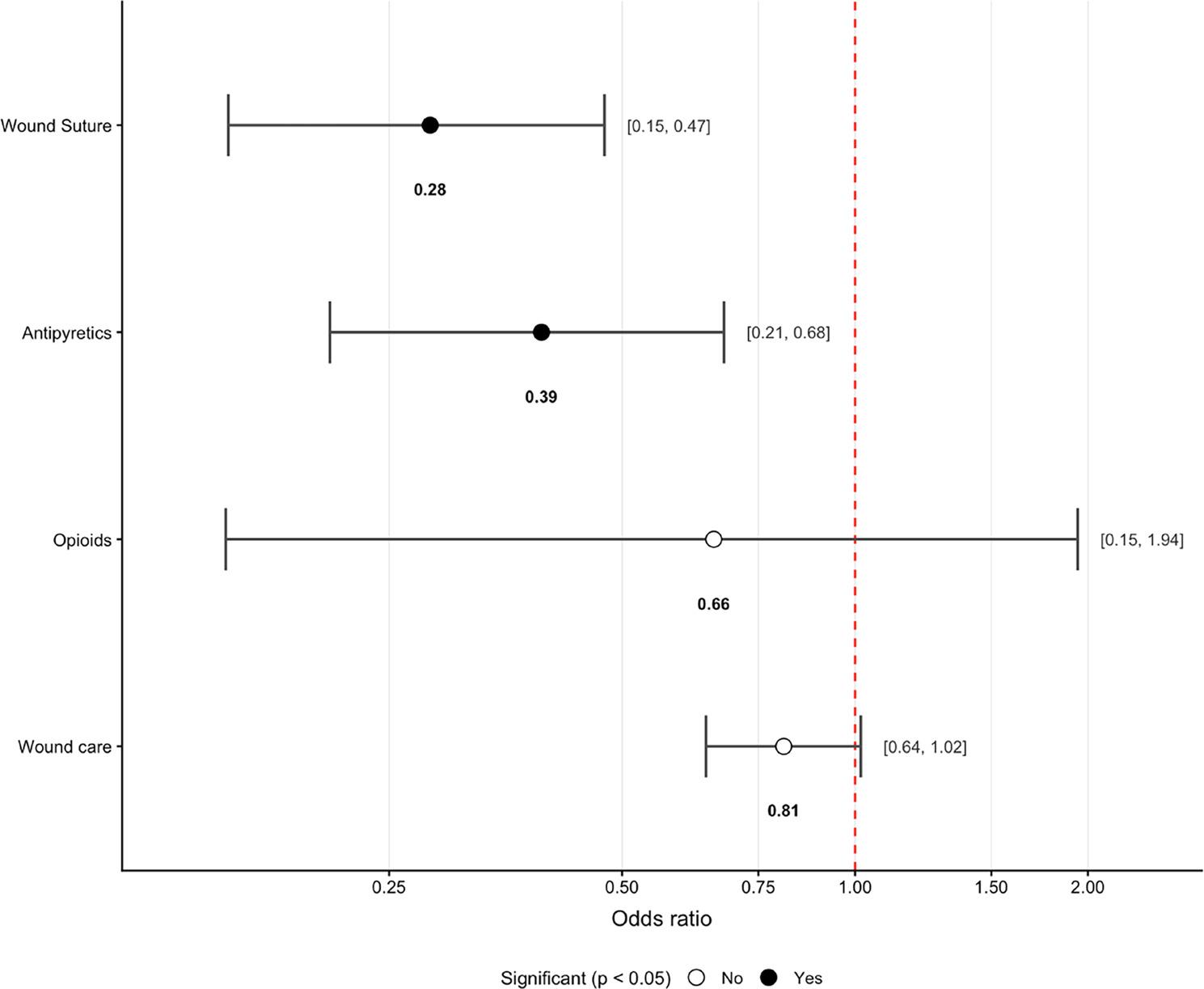
Odds Ratio with 95% CI of Interventions Negatively Associated with Referral

Wound suturing (OR 0.28; 95% CI 0.15–0.47) and antipyretic therapy (OR 0.39; 95% CI 0.21–0.68; both *p* < 0.01) were the strongest predictors of discharge (Fig. 6).

## Discussion

This is the first study of prehospital care during the current war in Gaza, offering critical insights into the functioning of one TSP in a high-intensity conflict. Despite the setting, the proportion of severely injured managed at the TSP was low, with minor injuries and non-trauma dominating. An overrepresentation of adult males is also notable. Unstable cases and those with fractures and penetrating injuries were significantly associated with referral, while non-traumatic cases and basic interventions predicted discharge. Corresponding interventions like airway management were more frequent in referred patients. This distribution reflects coherent triage. The TSP absorbed patients who would otherwise have presented directly to hospital, treating the majority on-site, selectively referring only those requiring a higher level of care. With 80.6% of patients discharged without hospital referral, the TSP likely reduced the number of cases reaching already overwhelmed facilities. By stabilizing the remaining patients who required referral, the TSP likely improved their condition before transport to hospital. The conservation of ambulance resources for evacuations follows from the same logic, by resolving most cases at point of contact at the TSP.

Unexpectedly for a TSP, more than half of all cases were non-traumatic, with infectious diseases dominating. Previous Mosul TSP data did not quantify non-trauma caseload but noted that non-trauma services expanded as battle progressed [16]. In effect, the TSP functioned as a primary care point rather than a trauma point. In many conflicts where health infrastructure collapses or becomes inaccessible, forward sites become default access points for primary care, as observed during the Syrian civil war [8]. .Internal displacement, disrupted continuity of care, and closure of regular health facilities likely shifted general medical needs onto the TSP.

Similar caseloads are not unique to conflict settings. Medical teams deployed following earthquakes encounter substantial proportions of non-trauma patients. Across these deployments, the proportion of earthquake-related diagnoses ranged from 28% to 67%, varying substantially by deployment timing and the availability of local medical infrastructure [26, 27]. These parallels suggest that forward-deployed medical units, regardless of disaster type, frequently evolve into primary care access points when surrounding health infrastructure is overwhelmed or destroyed.

Several factors may explain the dominance of minor injuries, non-trauma cases, and over-representation of adult males observed here. Severely injured patients likely die at point of injury before reaching the TSP due to inadequate, delayed or completely absent prehospital care. A similar observation of low in-hospital mortality during high-intensity conflict was previously reported in Mosul [28]. Other patients with severe injuries or women and children in general, may have bypassed the TSP entirely, directed by bystanders or ambulances directly to hospitals. Another potential explanation is that the location of the TSP was more accessible for the walking wounded, as anecdotally reported by staff deployed to this TSP. The restricted operating hours of the TSP may also have shaped patient attendance patterns, with non‑urgent cases more likely to present during daytime, while patients with urgent needs sought care at hospitals operating around the clock.

TSPs are intended for life-saving interventions close to point of injury [12, 15]. In Gaza, this was constrained by extreme conditions including direct Israeli attacks on healthcare, blockade on entry of medical supplies, and restrictions on medical evacuations [19, 29]. The Khan Younis TSP lacked patient tracking after referral and faced a wide spectrum of injuries and non-traumatic cases, limiting assessment of its broader impact. In contrast, Mosul TSPs operated within a semi-integrated emergency system under relatively safer conditions [13, 15]. Similarly, during the Gaza Mass Demonstrations in 2018–2019, TSPs were securely positioned, managing gunshot wounds and inhalations injuries exclusively [14]. Here instead basic care dominated, diverging sharply from resource-intensive military systems, where advanced care at point of injury, and rapid evacuation significantly reduced mortality in Afghanistan and Iraq [5, 6]. 

A precise quantification of lives saved by the Khan Younis TSP, or identification of the most beneficial interventions, is not feasible based on this dataset. Nevertheless, the observed high discharge rates and low referral rates are consistent with expected outcomes from stabilization and triage, even if it operated well below its trauma management capacity. Taken together, these findings suggest that the primary value of the Khan Younis TSP lay not in trauma management, but in functioning as a filter within a fragmented emergency system, meeting primary care demands and optimizing the use of scarce downstream resources.

Civilian TSPs, adhering to WHO standards, can provide a simplified model suited to low-security and resource-limited settings to deliver triage and stabilization. However, integration into an emergency system remains essential, as does the flexibility to adapt to local health needs.

### Further areas of research

Comparative studies across TSPs in Gaza and other conflict zones could identify best practices and operational bottlenecks. Referral tracking systems and outcome monitoring would enable more robust assessments of impact on survival and recovery. Qualitative studies with interviews of TSP-staff on working conditions, decision-making, and appropriateness of training and equipment are needed. Evaluating the cost-effectiveness of TSPs in different conflict settings would also inform strategic deployment and resource allocation.

Finally, care and mortality at point of injury need further study. To what extent should efforts focus on improving prehospital systems, on training lay people to act as 1st responders, and does this improve survival until the injured reach a hospital?

### Study limitations

Several study limitations must be acknowledged. First, the absence of data on pre-arrival interventions and transport modalities limits our understanding of patient flow and triage decisions. Second, the lack of data post-referral prevents assessment of both immediate and long-term outcomes, including survival and recovery. Third, the reliance on manual data entry and intermittent internet connectivity may have introduced reporting biases or data loss. For example, mass casualty incidents could overwhelm the documentation routines, leading to missing data.

## Conclusions

Despite the intended trauma focus and the high-intensity war setting of the Khan Younis TSP, the caseload was dominated by minor injuries and non-traumatic conditions. These patterns suggest that the Khan Younis TSP primarily functioned as a point for triage and primary care, likely offloading non-urgent patients from overwhelmed hospitals, and likely contributed in reducing consumption of limited ambulance resources.

The findings highlight the importance of adaptable, forward-positioned medical units in conflict zones, but also emphasize the need for improved integration, monitoring, and strategic planning.

## Data Availability

The data that support the findings of this study are available from Cadus e.V. but restrictions apply to the availability of this data, which were used under license for the current study, and so are not publicly available. Data is however available from the authors upon reasonable request and with permission of Cadus e.V.

## Abbreviations

EMT: Emergency medical team
IITT: Integrated interagency triage tool
PRCS: Palestinian red crescent society
TSP: Trauma stabilization point
WHO: World health organization
